# An automated simulator-independent workflow for reproducible simulation and analysis using Lancet and IPython Notebook

**DOI:** 10.1186/1471-2202-14-S1-P22

**Published:** 2013-07-08

**Authors:** Jean-Luc R Stevens, Marco Elver, James A Bednar

**Affiliations:** 1 Institute for Adaptive and Neural Computation, University of Edinburgh, EH8 9AB, UK; 2 Institute for Computing Systems Architecture, University of Edinburgh, EH8 9AB, UK

## 

Lancet [1] is a new, simulator-independent utility for succinctly specifying, launching, and collating results from large batches of interrelated simulations. Neural simulations require significant time and computational resources, particularly when exploring the large parameter spaces involved. Simulators rarely provide specific, comprehensive support for launching and collecting results across batch runs, and so the process of going from idea to publishable results typically involves an ad-hoc set of manual practices and/or one-off shell scripts. This informal process can be difficult to replicate later, because information about each of the processing steps is lost over time.

Here we demonstrate how Lancet can be used together with IPython Notebook [2] to provide a fully automated and fully reproducible workflow for neural simulations and similar batch-computing tasks. This workflow covers specifying what simulations are to be launched, storing metadata about each simulation run, collating the resulting output files, analyzing the results, and generating publication-quality figures that can be traced directly back to the original simulation and analysis code. This approach scales to hundreds of parallel jobs launched and simulation results spread across thousands of files, allowing users to focus on the scientific component of their work instead of writing repetitive boilerplate code.

Lancet is most useful with batch schedulers such as Oracle Grid Engine or other computing clusters, but also works well with single workstations. Users are given a small set of composable primitives that can succinctly specify large parameter spaces, from which individual jobs are generated. The declared simulation can then be reviewed in detail, avoiding mistakes before valuable time and computational resources are expended. All Lancet components are designed as self-contained, declarative objects that constitute the elements of a small DSL (domain specific language).

Once all the simulations are complete and the necessary files have been generated, Lancet collates the results for further analysis. To complete the workflow, the results can then be imported into an IPython Notebook, where they can be visualized interactively, with immediate feedback and a record of the analysis steps for reproducibility. This workflow allows you to assess your results for each simulation or compare results between different simulations. The generated data can be viewed in manageable chunks, without needing to directly manipulate files on either the local or remote filesystem. As parameters associated with each simulation are automatically recorded and tracked, all the relevant parameters are available for each file viewed. You can then process your data, saving it back out to separate files or to a database backend (HDF5 format using PyTables is currently supported [3]) while maintaining all the relevant metadata.

The core of Lancet is written in pure Python (Python 2 and 3 are supported), offering a general framework that is easily integrated with external tools and simulators that keeps track of all parameters used, ensuring a reproducible workflow. The fundamental design is entirely independent of the tools that are invoked, making Lancet a flexible and general tool for anyone who needs to run and analyze the data generated by hundreds of time-consuming simulations.
